# Efficacy of the Synbiotic Supplementation on the Metabolic Factors in Patients with Metabolic Syndrome: A Randomized, Triple-Blind, Placebo-Controlled Trial

**DOI:** 10.1155/2022/2967977

**Published:** 2022-04-16

**Authors:** Fatemeh Rahimi, Yahya Pasdar, Mojtaba Kaviani, Soheil Abbasi, Hillary Fry, Azita Hekmatdoost, Omid Nikpayam, Golbon Sohrab, Mansoaur Rezaei, Seyed Mostafa Nachvak, Reza Mohammadi

**Affiliations:** ^1^Nutritional Sciences Department, Kermanshah University of Medical Sciences, Kermanshah, Iran; ^2^School of Nutrition and Dietetics, Acadia University, Wolfville, NS, Canada; ^3^Department of Clinical Nutrition and Dietetics, Shahid Beheshti University of Medical Sciences, Tehran, Iran; ^4^Student Research Committee, Tabriz University of Medical Sciences, Tabriz, Iran; ^5^Department of Clinical Nutrition, Tabriz University of Medical Sciences, Tabriz, Iran; ^6^Department of Biostatistics, Medical School, Kermanshah University of Medical Sciences, Kermanshah, Iran; ^7^Food Sciences Department, Kermanshah University of Medical Sciences, Kermanshah, Iran

## Abstract

**Background:**

Metabolic syndrome is overwhelmingly increasing and is a significant risk factor for cardiovascular disorder, so effective treatment strategies are considered high priority. This study aimed to determine the effects of synbiotic supplementation on metabolic factors in patients with metabolic syndrome.

**Methods:**

In this triple-blind, randomized, placebo-controlled, clinical trial, 108 participants were divided into two groups to receive synbiotic supplementation or placebo for 12 weeks. All participants were also educated about maintaining a healthy lifestyle and consuming low-calorie nutritious meals, along with dietary intake and physical activity monitoring. Anthropometric measures, blood pressure, glycemic indices, lipid profile, hepatic enzymes, and hs-CRP were evaluated at the baseline and end of the trial.

**Results:**

Synbiotic supplementation significantly reduces fasting blood glucose (FBG) levels in the intervention group versus placebo group [−14.69 ± 15.11 mg/dl vs. −8.23 ± 7.90 mg/dl; *p*=0.007], but there was no difference between groups in other metabolic factors.

**Conclusions:**

These findings suggest that synbiotic supplementation while following a healthy lifestyle and nutrition improved FBG in patients with metabolic syndrome.

## 1. Introduction

Metabolic syndrome (MetS) is defined as a cluster of risk factors including lipid abnormalities, insulin resistance (IR), and abdominal obesity [1] leading to chronic diseases such as cardiovascular diseases (CVDs) and type 2 diabetes mellitus (T2DM) [2]. The World Health Organization (WHO) estimates that 300 million people worldwide will have developed MetS and its related complications by 2025 [3]. Measures to control MetS impose a great expense on the health system of a country, yet figures vary in terms of race and ethnicity in different populations [4].

Elevated blood glucose levels and insulin resistance (IR) are two main disorders in individuals with MetS [5] that lead to CVDs and T2DM if uncontrolled and untreated [2]. There is also a notable and strong association between MetS and nonalcoholic fatty liver disease (NAFLD) [6]. Lipid abnormalities including hypertriglyceridemia and low concentration of high-density lipoprotein cholesterol (HDL-C) are also characteristics of MetS [7] which, in combination with high plasma low-density lipoprotein cholesterol (LDL-C) and total cholesterol (TC), play a major role in developing CVDs [2].

Being overweight and having truncal obesity are pivotal features of MetS and are highly associated with IR and beta-cell dysfunction which are the risk factors associated with T2DM and CVDs. Similarly, developing secondary hypertension is a common problem faced by patients with T2DM and CVDs, but early diagnosis and treatment can prevent macrovascular diseases and microvascular complications such as renal diseases and diabetic neuropathy [8]. Recent studies suggest that the high prevalence of obesity and T2DM is due to not only genomic alteration, food habits, and sedentary lifestyle, but also gut microbiota, which may play a fundamental role in this issue [9]. Various reasons including poor diet, stress, aging, different types of infections, and antibiotic therapy can disrupt balance of gut microbiota and lead to dysbiosis, an imbalance of gut ecosystem [10].

Synbiotics are a combination of probiotics and prebiotics that exert their effects together creating enhanced effects, including improvement in diarrhea caused by antibiotic therapy, mitigation of constipation issues, alleviation of inflammatory diseases of gastrointestinal tract (GI), and improvement in lipid profiles [11]. Probiotics are healthy microorganisms which are naturally acquired from food and colonize our gut, particularly in the large intestine, and perform a variety of beneficial functions for human health, like having a positive influence on metabolism of lipids and glucose, the amount of lipid storage in liver cells, and immune system that overall assist individuals to lose weight and achieve metabolic balance [12, 13]. Prebiotics are nondigestible food components such as oligosaccharides and inulin that are transferred to the gut in intact forms and are fermented to short chain fatty acids (SCFAs) by gut microbiome, particularly bifidobacteria and lactobacilli, creating an energy source that promotes growth of these helpful probiotic species in a synergistic way [11].

It has been proposed that synbiotics have promising effects on ameliorating MetS indices [14]. Therefore, we conducted this triple-blind, randomized, placebo-controlled, clinical trial (RCT) to determine the impact of synbiotic supplementation on individuals with MetS.

## 2. Materials and Methods

### 2.1. Participants

This randomized, triple-blind, placebo-controlled, clinical trial was conducted in Kermanshah, Iran, from November 2016 to March 2017. The estimated sample size was 55 subjects for each group to detect 40 mg/dl difference in the fasting blood glucose (FBG); a power (1–*β*) of 80% and *α* = 0.05 were also used in the study. Although considering the possibility of attrition, we added about 20% to the primary estimated number. In total, 65 subjects with MetS were assigned to each group. The mean and standard deviation were obtained from the study by Shakeri et al. [15]. To recruit, the trial was announced during a number of presentations for employees of organizations in the city, a poster was shared on popular social media channels, and finally potential subjects were contacted both face to face and on the phone. Screened individuals who met the inclusion criteria attended our laboratory in the School of Nutritional Sciences and Food Technology to participate in the study. Moreover, informed written consent was acquired from all participants before enrolling in the study.

Based on the aforementioned definition presented by the NCEP ATPIII, those who had at least 3 items of the following criteria had been diagnosed with MetS and were eligible to participate in the study: waist circumference ≥88 cm for women and ≥102 cm for men; triglycerides (TG) ≥ 150 mg/dL, HDL-C ≤ 50 mg/dL for women and ≤40 mg/dL for men; blood pressure (BP) ≥130/85 mmHg; and fasting blood glucose (FBG) ≥ 100 mg/dL [16]. Exclusion criteria consisted of chronic diseases such as diabetes; kidney and liver abnormalities; tuberculosis; history of cardiovascular surgery; noncompliance with protocol; initiating medication use which would affect the investigated factors; some medicines such as oral contraceptives, estrogen, progesterone, and corticosteroids; pregnancy or lactation; using medication to control BG, blood lipid, and BP; insulin therapy; concurrent antibiotic use; probiotic supplements; or eating foods containing probiotics within 1 month before entering, or throughout, the trial.

### 2.2. Study Design

Subjects were randomized into synbiotic (*n* = 65) or placebo (*n* = 65) groups. Random allocation was conducted using block randomization technique [17]. Subjects in the intervention group received 2 capsules containing 500 mg (total dosage of 1000 mg) of synbiotic (FamiLact^®^, Zist Takhmir Co., Tehran, Iran) per day, and subjects of the placebo group received similar dosage of placebo capsules (starch 75%, lactose 22%, magnesium stearate 1%, silicon dioxide 1%, talc 1%) to consume after breakfast and lunch for 12 weeks. Active synbiotic supplements and placebo capsules were identical in terms of packaging, appearance, weight, color, and odor. Synbiotic capsules contained *Lactobacillus casei*, *Lactobacillus rhamnosus*, *Lactobacillus acidophilus*, *Lactobacillus bulgaricus*, *Bifidobacterium longum*, *Bifidobacterium breve*, and *Streptococcus thermophiles*; probiotic blend; 10^9^ CFU. In addition, prebiotic was short chain fructooligosaccharide (FOS), and each capsule included 38.5 g FOS (7.7% of each capsule). Other ingredients included lactose, magnesium stearate, and talc, and both active and placebo capsules were gluten-free. Patients received half of their capsules at the beginning of the study and the remainder in the middle of the trial (45^th^ day). We also asked them to return the capsule sheets, either fully consumed or not, to check compliance. If less than 80% of the capsules had been taken, they were excluded from the study. At the outset of the trial, we prepared educational packages consisting of brochures, group oral presentations, and individual dietary counseling. Patients were educated about principles of a healthy lifestyle, the food pyramid and MyPlate [18], how to choose and purchase nutritious foods, and various methods of cooking to prepare appealing, low-calorie meals. The study design was based on the Declaration of Helsinki, approved by the Ethics Committee of the Deputy of Research and Technology of Kermanshah University of Medical Sciences (Ethical Approval No. KUMS.REC.1395.467), and registered with the Iranian Clinical Trials Registry (Identifier: IRCT201608299856N3) (https://en.irct.ir/trial/10392).

### 2.3. Dietary Intake and Physical Activity Monitoring

A 3-day dietary recall was gathered at the beginning and at the end of the study to assess dietary intake. Food consumption was monitored via phone every 10 days to ensure the adherence to the given diets. We used Nutritionist IV software (First Databank, San Bruno, CA) for Iranian foods, to assess the nutrient intake of participants. Participants were also instructed to avoid eating yogurt and kefir and to substitute them with other types of dairy products to fulfill their calcium and protein needs. The short form of the international physical activity questionnaire (IPAQ) [19] was used to evaluate physical activity as total metabolic equivalent task-hour/day (MET-h/d). Moreover, we asked subjects to maintain their level of activity during the trial. Additionally, participants had access to researchers via phone call and/or attending the laboratory if questions were to arise.

### 2.4. Anthropometric and Blood Pressure Assessments

We measured anthropometric indices including body weight, lean body mass (LBM), body protein, and body fat using Body Analyzer, model “Jawon Medical Avis 333 Plus” (Korea). Standing height was measured by stadiometer (Germany), brand: Seca, model “220, CE0123.” Body mass index (BMI) was calculated as weight (kg)/height (m)^2^. Waist, a narrowest area below the rib cage and above the umbilicus and hip, and the largest area below the waist were measured using an inelastic tape measure. Systolic blood pressure (SBP) and diastolic blood pressure (DBP) were measured using a digital brachial sphygmomanometer, brand: Omron (Vietnam), after sitting for 20 min in a quiet environment. All measures were conducted at the beginning and at the end of the study.

### 2.5. Biochemical Assessments

Fasting blood (10 ml) was withdrawn from the brachial vein in the morning at 7.30–9.30 after 8–10 hours of fasting by a trained phlebotomist at the beginning and after 12 weeks. All blood samples were collected in tubes containing EDTA gel to protect them from lysing and were centrifuged at 4000 rpm for 5 min using Universal 320 R centrifuge (Germany) to separate plasma from whole blood. Afterwards, separated plasmas were poured into micro tubes and quickly transferred to −40 centigrade degree freezer. At the end of 12-week period, plasmas were moved to the laboratory to measure biochemical factors including FBG, insulin, HDL-C, TC, and TG. LDL-C concentration was also calculated using Friedewald formula: LDL = TC −HDL− (TG/5) [20]. FBG, lipid factors, and hepatic enzymes including serum glutamate-pyruvate transaminase (SGPT), serum glutamic oxaloacetic transaminase (SGOT), and alkaline phosphatase (ALP) were measured using enzymatic calorimetric method via autoanalyzer, model: Mindray BS-38, Germany, with kits from Pars Azmoon (Tehran, Iran). The measurement of high-sensitivity C-reactive protein (hs-CRP) was conducted using the same method through COBAS INTEGRA 400 plus/800 analyzer with kit from Monobind (the USA). Measurement of insulin was conducted using Chemiluminescence/ELISA method via Denka Seiken (Tokyo, Japan) with kit from Monobind (the USA). HOMA-IR and HOMA-*β* were calculated as [FBG (mmol/L) × fasting insulin (mU/L)/22.5] and [fasting insulin levels (*μ*U/ml) × 20/[FBG (mmol/L) − 3.5]], respectively [21, 22]. IGR was calculated as [fasting insulin (mU/L)/FBG (mg/dL)]

### 2.6. Statistical Assessments

In the present study, data were analyzed using Statistical Package for Social Sciences version 16 (SPSS Inc., Chicago, IL, USA). The ANCOVA test was used to control confounding factors. Quantitative variables were expressed by mean ± standard deviation (SD), and qualitative variables were indicated in the form of percent and frequency. Data normality was analyzed through Kolmogorov–Smirnov test. We used an independent *t*-test in order to compare results between case and control group for normal variables, while Mann–Whitney *U* test was used to make comparisons between the two groups for nonnormal variables. Additionally, considering each group separately for comparing data before and after intervention, paired samples *t*-test was applied for normal results and Wilcoxon test for nonnormal ones. Significant level was set as *p* < 0.05.

## 3. Results

**Figure 1:**
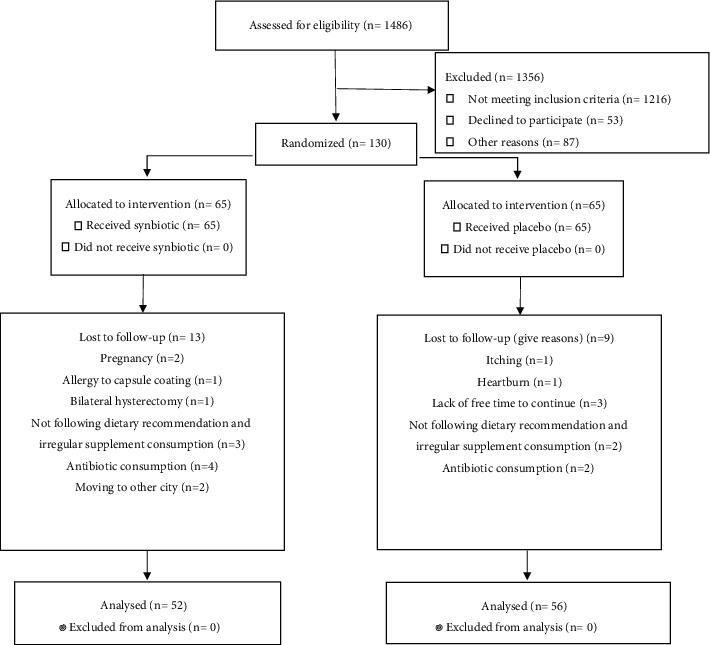
Flow diagram of the trial participants.

A total of 130 subjects participated in the study (65 subjects in the synbiotic group and 65 subjects in the placebo group), and 108 participants completed the trial (52 subjects in the synbiotic group and 56 subjects in the placebo group). Pregnancy, allergy to capsule coating, bilateral hysterectomy, irregular supplement consumption, antibiotic therapy, migrating to other city, heartburn, itching, and lack of free time to continue the trial period were reasons for discontinuing the trial. The baseline characteristics of the trial participants are shown in Table 1. Based on Table 1, there were no significant differences between the two groups in terms of age and gender distribution, weight, educational status, and physical activity before and after the trial (*p* > 0.05) (see Figure 1).

As Table 2 illustrates, energy intake of participants from the designed diet was about 1400 kcal/day, with glucose and saturated fatty acid restriction. As can be inferred from Table 2, intake of energy, glucose, and other nutrients showed a significant decline in within-group analysis (<0.001), yet there were no significant changes between groups after the intervention (*p* > 0.05 for all). Analysis of covariance did not show any significant difference in dietary intake (*p* > 0.05 for all).

Mean and standard deviations of anthropometric measures and blood pressure are presented in Table 3. Between-group analysis did not show any significant difference in weight, BMI, WC, HC, WHR, LBM, body protein, body fat, SBP, and DBP at the baseline and end of the trial (*p* > 0.05 for all). According to the within-group analysis, weight, BMI, WC, HC, WHR, SBP, body protein, and body fat have been declined in both groups (*p* < 0.05 for all), but LBM in both groups and DBP in the synbiotic group did not significantly change. Analysis of covariance demonstrated that synbiotic supplementation has not had any effect on anthropometric indices and blood pressure (*p* > 0.05 for all).

However, between-group analysis after the trial compared to the baseline showed a significant reduction of FBG (*p* > 0.007) (Table 4). As shown in Table 4, HOMA-*β* is increased in both groups at the end of trial in comparison to the baseline (*p* < 0.05), but between-group analysis did not show significant difference. IGR had increased only in synbiotic group in comparison to the baseline, although within-group comparison did not demonstrate any difference (*p* > 0.05). From Table 4, we can also see that TG had fallen significantly in the synbiotic and placebo groups (*p*=0.001 for both) in within-group comparison. TC showed a significant decrease not only in within-group comparison in just the synbiotic group (*p*=0.007), but also in between-group analysis (*p*=0.047). In regard to lipoprotein cholesterols, LDL-C was just slightly improved, yet it was not significant neither in posttrial comparison (*p*=0.949) nor in comparison to the baseline for the synbiotic group (*p*=0.368) and placebo group (*p*=0.062). In contrast, HDL-C plasma concentration rose significantly in the placebo group (*p*=0.016) after the trial, whereas between-group analysis did not indicate a significant rise (*p*=0.503). Between- and within-group analysis indicated that there are not any significant changes in hs-CRP, SGOT, SGPT, ALP, and insulin (*p* > 0.05). According to the analysis of covariance, synbiotic supplementation had a beneficial effect only on FBG level but did not show any significant effects on the other biochemical factors in comparison to placebo.

## 4. Discussion

The outcomes of the current trial demonstrated that synbiotic supplementation caused a significant fall in FBG concentration but had no effects on insulin level, HOMA-IR, HOMA-*β*, and IGR. There are some studies that agree with our findings, for instance, a study that lasted 28 weeks on patients with MetS supplemented with two daily dosages of 2 × 10^8^ CFU of seven strains of probiotics combined with FOS, which also resulted in FBG decrease and had beneficial effects on insulin level and HOMA-IR [23]. Furthermore, another study on 120 prediabetic subjects for 24 weeks demonstrated a significant decline in hyperglycemia prevalence in probiotic and synbiotic groups compared to a group consuming placebo [24]. In contrast, the results of a trial performed on 81 subjects who developed diabetes and consumed synbiotic bread consisting of 1 × 10^8^ CFU *Lactobacillus sporogenes* and 0.07 gram inulin per one gram, three times a day for a period of 8 weeks [25], were not consistent with our findings in terms of FBG plasma level. This inconsistency may be due to several reasons, since the sample size and duration of this study were smaller than those of our trail, and participants of the trial were supplemented only with one strain of probiotics, while subjects of our study were taking synbiotic capsules containing seven strains. Therefore, it seems that synbiotics with multistrain probiotics would have more FBG lowering effects compared to single strain ones [26]. Some mechanisms explain how probiotics can positively affect blood glucose. For instance, *Lactobacillus plantarum* Ln4 (Ln4) can significantly reduce BG through stimulating glucose uptake in 3T3-L1 adipocytes, significantly decreasing insulin resistance index (HOMA-IR), and increasing oral glucose tolerance test (OGTT) and insulin response. Ln4 may work by changing the expression of some hepatic genes involved in glucose regulation by increasing those genes' mRNA levels [27]. Besides synbiotics, low-calorie diet can lead to a decline in fasting endogenous glucose production along with rise in secretion of insulin, which stems from improvement in *β* cell function. At the same time, a decrease in fasting glucagon levels results from decrement in *α*-cell secretion, considered an important factor in reducing FBG [28].

According to the statistical analysis, synbiotic supplementation did not have any significant effect on hs-CRP level. Some studies are in line with our result; for instance, a randomized controlled clinical trial in pregnant women could not find any significant effect of synbiotic supplementation on hs-CRP [29]. Another crossover controlled trial study showed that consumption of fortified synbiotic food for six weeks did not bring about any effect on hs-CRP in type 2 diabetes mellitus patients [30]. However, some studies showed results that are inconsistent with our findings. For example, the study by Zamani et al. indicated that synbiotic supplementation for 8 weeks significantly declined hs-CRP level [31]. Another clinical trial study also revealed that intake of synbiotic supplement for 12 weeks substantially reduced hs-CRP concentration [32]. The lack of synbiotic effects on hs-CRP in our study may be because the type of pre/probiotics, dosage of supplement, duration of study, and hs-CRP concentration of patients at the baseline of the study were not in the abnormal range.

In addition to the impact of synbiotics on hs-CRP, lipid profile has been affected by pro/pre/synbiotics [15]. Regarding the ability of synbiotics to improve plasma cholesterol, SCFAs are produced by poly- and oligosaccharides fermentation through probiotics that inhibit activation of HMG-CoA reductase (3-hydroxy-3-methyl-glutaryl-coenzyme A reductase), a key enzyme in pathway of cholesterol synthesis [33]. SCFAs can cease hepatic uptake of plasma cholesterol and block cholesterol production in liver as well [34]. One particularly salient example of this is butyrate and propionate: butyrate prevents hepatic cholesterol synthesis, and propionate decelerates speed of its synthesis [35, 36]. The results of our investigation showed that plasma levels of TC were significantly reduced in the synbiotic group when comparing the beginning and end of the trial. These findings are in accordance with some previous research [36, 37]. However, some studies could not demonstrate positive effects of synbiotics on TC. For example, a trial conducted on 90 pregnant women with gestational diabetes mellitus (GDM) for 6 weeks to survey the effect of synbiotic supplementation—consisting of *L. acidophilus*, *L. plantarum*, *L. fermentum*, and *L. gasseri* (1.5–7.0 × 10^9-10 ^CFU/g)—with fructooligosaccharide (38.5 mg) on insulin resistance and lipid profile did not show a significant reduction in TC plasma levels [38] which may be due to the shorter intervention period and physiological status of subjects who were pregnant. Regarding the mechanisms allowing synbiotics to improve TG plasma concentration, SCFAs which produced in the colon by beneficial bacteria in the body, especially lactate, lead to a decrease in TG concentration [39], some particular types of proteins secreted from intestine, and regulates lipid metabolism in peripheral organs, all of which have been effected by gut microflora. In fact, gut microflora has effects on expression of genes related to enterohepatic system metabolism [40]. A low-carbohydrate diet may lead to a fall in plasma TG through downregulation of hepatic de novo lipogenesis, elevate the amount of lipoprotein lipase (LPL) in muscles, and eventually increase clearance of TG [41]. Plasma TG levels of our subjects experienced significant attenuation in both groups. This outcome was nearly in parallel with a study conducted on 62 diabetic patients to determine the effect of synbiotic food consumption on their metabolic parameters, showing a significant reduction in serum TG levels. An RCT conducted on 277 diabetic patients with obesity or overweight—to compare low-fat, low-carbohydrate diet with the usual diet in terms of changes in CVDs risk factors—confirmed the significant reduction in TG level in those who followed low-calorie diet [42]. Nevertheless, some other studies were not in accordance with other research [36, 37]. These studies contained smaller sample size, and they were using single strain of bifidobacteria with FOS and two strains of *L. acidophilus* and *B. lactis* in yogurt. Of note, when using yogurt, the number of strains is fewer than those in a supplement, such as the one used in the present study.

Plasma LDL-C showed a minimal drop in both groups; this decline was a bit higher in synbiotic group, yet it was not significant. A trial conducting to compare effects of probiotic and conventional yogurt was in parallel with our study in some aspects; it showed a significant decline in LDL-C plasma in both groups [43], which was similar to our outcomes in terms of lowering effect in both groups and different from our results regarding being significant. In fact, the average plasma LDL-C levels in all of our participants were inserted in normal spectrum, so perhaps observing significant change in this proper LDL-C concentration is not probable or even not expected. In addition, results of a study on effects of synbiotic supplementation on individuals with MetS did not suggest a significant fall in LDL-C levels in the intervention group compared to the control group [23]. However, a trial carried out on 48 subjects with normal weight illustrated a significant reduction in LDL-C concentration followed by a 1-year calorie restriction [44]. Additionally, HDL-C level increased slightly in both groups maybe due to modifying food habit based on dieticians' consultation. Studies on effects of synbiotics on HDL-C are controversial: some claim that synbiotics increase HDL-C levels substantially [45], and others fail to indicate the significant positive impact [37]. Some studies suggest that increasing LPL level is attributed to low carbohydrates diet that can result in enhancement of catabolism of TG-rich lipoproteins that leads to creating HDL-C from nonesterified cholesterol, apoprotein, and phospholipid [41]. A review paper investigating the effect of calorie restriction on risk factors of CVDs confirmed that long-term low-calorie diet can greatly boost HDL-C level [46], while some studies could not find evidence denoting the positive impact of low-calorie diet on it [47]. Regarding decline in plasma concentration of TG, LDL-C, and HDL-C, we can propose that these changes may result from low-calorie, healthy diet to some extent that some studies representing positive effects of low-calorie diet on TG [42, 48], and originated from synbiotic supplementation as well, since the changes of this biochemical factors was greater in the synbiotic group versus control group.

Our study demonstrates that intake of synbiotic supplement did not any have significant impact on hepatic enzymes including SGOT, SGPT, and ALP. There are studies that have shown similar findings to our study. For example, a clinical trial indicated that intake of synbiotic supplement for 8 weeks did not produce any considerable effect on SGOT and SGPT [49]. In Mofidi et al.'s study on nonalcoholic fatty liver disease (NAFLD) patients, synbiotic supplementation for 28 weeks showed no effects on SGOT, SGPT, and ALP levels [50]. However, most of studies in this field had findings contradicting our studies [51–53]. This inconsistency may be because the level of hepatic enzymes in the participants in our study was in the normal range, although the hepatic enzymes showed a nonsignificant decreasing trend in the synbiotic group in comparison with the placebo group.

Some studies propose that antiobesity effects of probiotics, particularly *Lactobacillus* species, originated from regulation of lipid and glucose metabolism, leptin regulation, and decrement of size of adipocytes [54, 55]. Prebiotics also help to improve anthropometric indices such as waist circumference (WC) by positively affecting gut microbiota and enhancing growth and activity of probiotics [56]. In addition to synbiotics, calorie-restricted diet is considered as the first choice for weight loss, and some studies confirm that low-calorie diet results in weight loss, particularly decreasing central obesity [57]. Based on the outcomes of anthropometric indices of our trial, weight, BMI, WC, HC, and body fat showed an inclination to be significantly reduced in both groups at the end of trial in comparison the baseline. This finding perhaps can be considered as a proof to other studies that suggest the promising effects of healthy diet and modifying lifestyle on reducing visceral fat in both subjects with MetS and healthy population [58].

Blood pressure of all the subjects of this trial was within the normal range at baseline. The subjects of the two groups however showed a BP reduction after the intervention. Decrement of BP in all the participants may stem from following healthy diet principles including those related to combating hypertension. For example, following a diet rich in calcium results in improvement of vasoconstriction and decline in BP [59], and as all of our subjects were recommended to consume regular two servings of dairy products including milk [60] and not receiving other kinds of dairy products such as yogurt, yogurt drink, and kefir, this may had a slight reducing effect on BP in the two groups. Moreover, prebiotics enhance dietary calcium absorption through binding to calcium and transferring together to colon; then, calcium detaches from prebiotics and, by being located in an acidic environment made by SCFAs in distal colon, eventuates in more calcium concentration in colon and more absorption by colonocytes which assist blood pressure to be decreased [61]. Apart from prebiotic role in controlling hypertension, probiotic consumption produces nitric oxide (NO) which possesses a major role in vasodilation and consequently BP decrement [62].

This study contains some limitations. This trial was conducted in the winter season when contagious diseases such as influenza are more prevalent compared with other seasons. Some subjects needed to consume antibiotics, because of influenza or having a cold, which was one of excluding criteria. Moreover, synbiotic capsules should be kept in refrigerator and could not be carried out of home, which resulted in irregular consumption in some of our subjects.

## 5. Conclusions

Based on the result of our trial, consumption of synbiotics, in combination with education about healthy lifestyle and nutrition, results in FBG reduction in comparison with placebo. In fact, modifying gut microbiota along with calorie restriction leads to improvement of MetS indices.

## Figures and Tables

**Table 1:** Baseline characteristics of the trial participants.

Variable	Synbiotic group (*n* = 52)	Placebo group (*n* = 56)	*p* value
Age (year)	42.77 ± 8.35	45.64 ± 9.33	0.096^*∗*^
Gender (%)			0.482^#^
Female	57.7	64.3	
Male	42.3	35.7	
Educational status (%)			0.173^#^
Under diploma	3.8	12.5	
Diploma	17.3	23.2	
Bachelor	65.4	58.9	
Master's and higher	13.5	5.4	
Total MET-h/d			
Baseline	1160.91 ± 1173.12	1538 ± 1104.90	0.099^*∗*^
At 12 wk	1122.98 ± 1132.31	1550.14 ± 1132.31	0.061^*∗*^

Data are reported as mean ± SD or number (percent). *p* value less than 0.05 is considered significant; *p* value^*∗*^: calculated using independent-samples *t*-test; *p* value^#^: calculated using chi-square. MET-h/d: metabolic equivalent task-hour/day.

**Table 2:** Daily dietary intake of participants during the trial (mean ± SD).

Variables		Mean ± SD		*p* value^2^	*p* value^3^
		Synbiotics (*n* = 52)	Placebo (*n* = 56)		
Energy (kcal)	Baseline	1963.75 ± 437.99	1903.82 ± 368.23	0.578	
	At 12 wk	1443.86 ± 290.36	1526.90 ± 440.42	0.382	
	*p* value^1^	<0.001	<0.001		0.21

Protein (g)	Baseline	73.27 ± 27.69	70.95 ± 17.95	0.702	
	At 12 wk	55.13 ± 24.10	64.10 ± 20.68	0.137	
	*p* value^1^	0.02	0.11		0.16

Fat (g)	Baseline	80.09 ± 32.18	77.22 ± 24.23	0.703	
	At 12 wk	51.74 ± 8.79	54.99 ± 23.43	0.578	
	*p* value^1^	<0.001	0.001		0.49

Carbohydrate (g)	Baseline	242.38 ± 56.58	237.52 ± 58.43	0.755	
	At 12 wk	188.53 ± 50.42	195.78 ± 62.46	0.642	
	*p* value^1^	<0.001	<0.001		0.36

Glucose (g)	Baseline	54.49 ± 18.28	59.75 ± 19.59	0.308	
	At 12 wk	33.70 ± 16.43	35.21 ± 22.73	0.783	
	*p* value^1^	<0.001	<0.001		0.59

*p* value^1^: significance of within-group alterations (paired *t*-test); *p* value^2^: significance of between-group alterations (independent *t*-test); *p* value^3^: treatment by analysis of covariance (adjusted baseline variable).

**Table 3:** Anthropometric indices and blood pressure before and after the trial (mean ± SD).

Variables		Mean ± SD		*p* value^2^	*p* value^3^
		Synbiotics (*n* = 52	Placebo (*n* = 56)		
Weight (kg)	Baseline	84.28 ± 14.92	82.04 ± 14.24	0.503	
	At 12 wk	82.12 ± 14.29	79.43 ± 13.55	0.325	
	*p* value^1^	<0.001	<0.001		0.420

BMI	Baseline	31.03 ± 4.91	31.14 ± 5.33	0.91	
	At 12 wk	30.67 ± 4.73	30.62 ± 5.29	0.84	
	*p* value^1^	<0.001	<0.001		0.31

WC (cm)	Baseline	104.84 ± 9.74	104.60 ± 9.99	0.900	
	At 12 wk	99.49 ± 8.98	99.53 ± 10.38	0.981	
	*p* value^1^	<0.001	<0.001		0.640

HC (cm)	Baseline	108.86 ± 9.90	109.28 ± 10.22	0.82	
	At 12 wk	104.50 ± 9.07	104.38 ± 9.06	0.94	
	*p* value^1^	<0.001	<0.001		0.56

WHR	Baseline	0.93 ± 0.06	0.94 ± 0.06	0.78	
	At 12 wk	0.90 ± 0.06	0.91 ± 0.05	0.52	
	*p* value^1^	<0.001	<0.001		0.58

SBP (mmHg)	Baseline	118.98 ± 12.94	120.85 ± 14.23	0.486	
	At 12 wk	114.19 ± 11.31	113.76 ± 10.30	0.841	
	P value^1^	0.039	<0.001		0.272

DBP (mmHg)	Baseline	81.22 ± 8.7	81.94 ± 9.45	0.686	
	At 12 wk	79.30 ± 8.85	78.29 ± 7.14	0.530	
	*p* value^1^	0.147	0.014		0.480

LBM (kg)	Baseline	54.72 ± 11.30	52.82 ± 9.55	0.34	
	At 12 wk	55.10 ± 11.31	52.69 ± 9.16	0.23	
	*p* value^1^	0.55	0.78		0.75

Body proteins (kg)	Baseline	10.52 ± 2.38	10.11 ± 2.00	0.33	
	At 12 wk	10.57 ± 2.34	10.16 ± 2.05	0.34	
	*p* value^1^	0.04	0.03		0.98

Body fat (%)	Baseline	35.53 ± 5.76	35.94 ± 6.37	0.72	
	At 12 wk	33.81 ± 6.40	33.74 ± 7.26	0.95	
	*p* value^1^	<0.001	<0.001	0.39	

*p* value^1^: significance of within-group alterations (paired *t*-test); *p* value^2^: significance of between-group alterations (independent *t*-test); *p* value^3^: treatment by analysis of covariance (adjusted baseline variable). WC: waist circumference; BMI: body mass index; HC: hip circumference; WHR: waist–hip ratio; LBM: loan body mass SBP: systolic blood pressure; DBP: diastolic blood pressure.

**Table 4:** Biochmistry variables before and after the trial (mean ± SD).

Variables		Mean ± SD		*p* value^2^	*p* value^3^
		Synbiotics (*n* = 52)	Placebo (*n* = 56)		
FBG (mg/dl)	Baseline	103.37 ± 22.92	97.60 ± 16.92	0.150	
	At 12 wk	88.67 ± 13.52	89.38 ± 14.96	0.804	
	*p* value^1^	<0.001	<0.001		0.007

Insulin (*μ*M/ml)	Baseline	6.73 ± 2.93	6.54 ± 2.94	0.75	
	At 12 wk	7.66 ± 3.76	7.36 ± 3.70	0.68	
	*p* value^1^	0.17	0.28		0.88

HOMA-IR	Baseline	1.73 ± 0.82	1.58 ± 0.74	0.35	
	At 12 wk	1.72 ± 0.85	1.60 ± 0.77	0.43	
	*p* value^1^	0.98	0.94		0.95

HOMA-*β*	Baseline	72.82 ± 46.76	79.77 ± 47.98	0.463	
	At 12 wk	133.17 ± 90.40	126.59 ± 88.68	0.711	
	*p* value^1^	<0.001	0.001		0.41

IGR	Baseline	1.21 ± 0.57	1.24 ± 0.58	0.806	
	At 12 wk	1.62 ± 0.78	1.54 ± 0.84	0.641	
	*p* value^1^	0.005	0.06		0.64

Hs-CRP (mg/dl)	Baseline	2.28 ± 1.91	2.82 ± 2.95	0.57	
	At 12 wk	2.25 ± 2.31	2.31 ± 1.91	0.27	
	*p* value^1^	0.93	0.31		0.89

SGPT (mg/dl)	Baseline	24.35 ± 13.72	20.96 ± 11.67	0.18	
	At 12 wk	22.71 ± 11.49	21.85 ± 10.06	0.68	
	*p* value^1^	0.24	0.44		0.16

SGOT (mg/dl)	Baseline	25.41 ± 7.87	25.06 ± 11.32	0.85	
	At 12 wk	23.08 ± 6.75	24.15 ± 11.46	0.57	
	*p* value^1^	0.01	0.30		0.26

ALP (mg/dl)	Baseline	184.08 ± 50.33	198.58 ± 78.33	0.27	
	At 12 wk	180.10 ± 53.72	199.34 ± 82.14	0.16	
	*p* value^1^	0.16	0.79		0.24

TG (mg/dl)	Baseline	204.31 ± 111.01	198.58 ± 87.01	0.772	
	At 12 wk	160.92 ± 63.40	164.30 ± 75.84	0.808	
	*p* value^1^	0.001	<0.001		0.536

TC (mg/dl)	Baseline	224.41 ± 38.04	237.87 ± 44.12	0.103	
	At 12 wk	214.47 ± 38.46	229.85 ± 38.72	0.047	
	*p* value^1^	0.007	0.062		0.730

LDL-C (mg/dl)	Baseline	93.84 ± 21.77	102.47 ± 21.00	0.044	
	At 12 wk	92.12 ± 20.06	100.57 ± 18.38	0.029	
	*p* value^1^	0.368	0.413		0.949

HDL-C (mg/dl)	Baseline	39.33 ± 8.98	39.13 ± 7.46	0.905	
	At 12 wk	40.33 ± 8.48	40.81 ± 6.02	0.738	
	*p* value^1^	0.195	0.016		0.503

*p* value^1^: significance of within-group alterations (paired *t*-test); *p* value^2^: significance of between-group alterations (independent *t*-test); *p* value^3^: treatment by analysis of covariance (adjusted baseline variable). FBG: fasting blood glucose; TG: triglycerides; TC: total cholesterol; LDL-C: low-density lipoprotein cholesterol; HDL-C: high-density lipoprotein cholesterol; HOMA-IR: homoeostatic model assessment-insulin resistance; HOMA-*β*: homoeostatic model assessment-beta; IGR: insulin–glucose ratio; hs-CRP: high-sensitivity C-reactive protein; SGPT: serum glutamate-pyruvate transaminase; SGOT: serum glutamic oxaloacetic transaminase, ALP: alkaline phosphatase.

## Data Availability

The datasets used and analyzed during the current study are available from the corresponding authors on reasonable request.
